# Effect of antivenom therapy of *Rhabdophis tigrinus* (Yamakagashi snake) bites

**DOI:** 10.1186/s40560-014-0044-5

**Published:** 2014-07-31

**Authors:** Toru Hifumi, Atsushi Sakai, Akihiko Yamamoto, Masahiro Murakawa, Manabu Ato, Keigo Shibayama, Hiroshi Kato, Yuichi Koido, Junichi Inoue, Yuko Abe, Kenya Kawakita, Masanobu Hagiike, Akihiko Ginnaga, Yasuhiro Kuroda

**Affiliations:** Emergency Medical Center, Kagawa University Hospital, 1750-1 Ikenobe, Miki Kita, Kagawa 761-0793 Japan; The Japan Snake Institute, Yabutsuka 3318, Ota Gumma, 379-2301 Japan; Department of Bacteriology II, National Institute of Infectious Disease, Gakuen 4-7-1, Musashimurayama-shi, Tokyo 208-0011 Japan; Department of Internal Medicine, Kaizuka Hospital, Hakosaki 7-7-27, Higashi-ku, Fukuoka 812-0053 Japan; Department of Immunology, National Institute of Infectious Disease, Toyama 1-23-1, Shinjuku-ku, Tokyo 162-8640 Japan; Division of Critical Care Medicine and Trauma, National Hospital Organization Disaster Medical Center, 3256 Midori-cho, Tachikawa, Tokyo 190-0014 Japan; Division of Critical Care Medicine and Trauma, Yamanashi Prefectural Central Hospital, 1-1-1 Fujimicho, Kofu, Yamanashi 400-8506 Japan; The Chemo-Sero-Therapeutic Research Institute (KAKETSUKEN), 1-6-1 Okubo, Kita-ku, Kumamoto-shi, Kumamoto 860-8568 Japan

**Keywords:** Yamakagashi, *Rhabdophis tigrinus*, Antivenom

## Abstract

**Background:**

*Rhabdophis tigrinus* (Yamakagashi snake) is a rear-fanged colubrid snake present throughout Russia and Asia. Its venom induces life-threatening hemorrhagic symptoms and severe disseminated intravascular coagulation with a fibrinolytic phenotype.

*R. tigrinus* antivenom manufactured by the immunization of horses to neutralize the venom has the risk of adverse events such as anaphylaxis and serum sickness disease. It should be used when benefit is greater than the risk of adverse effects; however, its efficacy has not been well evaluated.

Although our previous survey of nine cases demonstrated that seven of all cases treated with antivenom survived, the clinical characteristics and prognosis without antivenom administration remained unclear. We assumed that *R. tigrinus* antivenom administration overlaps self-recovery with supportive care. We aimed to determine the association between antivenom administration and outcome with further analyzed cases.

**Methods:**

We retrospectively reviewed the records of the Japan Snake Institute between January 1, 1973 and December 31, 2013. Antivenom and without antivenom groups were compared with regard to baseline demographic features, treatment-related factors, and outcomes.

**Results:**

In total, 34 patients were analyzed (97% male, median age 37.5 years). Twenty-five patients were further examined from our previous study. On admission, the median levels of fibrinogen and fibrinogen degradation products were 35 mg/dL and 200 μg/mL, respectively, and platelet counts were 107,000/mm^3^. The median disseminated intravascular coagulation score (defined by the Japanese Association of Acute Medicine) was 5. Antivenom was administered to 19 patients, with a median interval of 32 h between bite and antivenom administration. The in-hospital mortality rate was 12%. In univariate analysis, baseline characteristics and laboratory data were not significantly different between the antivenom and without antivenom groups. Hospital mortality in the antivenom group was significantly better than that in the without antivenom group (0% vs. 26.7%, *P* = 0.03). Moreover, the number of patients developing renal failure requiring hemodialysis was significantly lower in the antivenom group (5.3% vs. 40.0%, *P* = 0.03).

**Conclusions:**

In our small retrospective study, antivenom administration was likely to be effective in the management of *R. tigrinus* bites. Apparently, further research is required to confirm its efficacy.

## Background

*Rhabdophis tigrinus* (Yamakagashi snake) is a rear-fanged venomous snake present throughout Russia and Asia [1]. Its venom induces life-threatening hemorrhagic symptoms and severe disseminated intravascular coagulation (DIC) with a fibrinolytic phenotype [2].

*R. tigrinus* antivenom manufactured by the immunization of horses to neutralize the venom has the risk of adverse events such as anaphylaxis and serum sickness disease [1,2]. It should be used when benefit is greater than the risk of adverse effects; however, its efficacy has not been well evaluated. Although our previous survey of nine cases demonstrated that seven of all cases treated with antivenom survived, the clinical characteristics and prognosis without antivenom administration remained unclear [3]. Further, theoretically, *R. tigrinus* antivenom only neutralizes the unbound venom and cannot restore organ function. Antivenom was administered after patients developed severe DIC in the study (the median interval between bite and antivenom administration was 35 h) [2]. We assume that the *R. tigrinus* antivenom administration overlaps self-recovery with supportive care.

The present study therefore aimed to determine the association between antivenom administration and outcome with further analyzed cases.

## Methods

The institutional review board of the Japan Snake Institute approved the present study.

### Patients and setting

The Japan Snake Institute was established in 1968 to research medical application of snakes.

In clinical practice, physicians managing patients with snake bites usually ask for the assistance of the Japan Snake Institute, where diagnosis is confirmed according to laboratory data and clinical symptoms. Clinical data was routinely collected, and all cases of *R. tigrinus* bites were recorded in this institute. The records of the Japan Snake Institute were retrospectively investigated between January 1, 1973 and December 31, 2013.

### Diagnosis of *R. tigrinus* bites

*R. tigrinus* bites were diagnosed based on the detailed information of snakes that patients observed and hemorrhagic symptoms including severe hypofibrinogenemia, and final diagnosis was recorded in a file of the Japan Snake Institute.

We also applied DIC diagnostic criteria for critically ill patients, as outlined by the Japanese Association of Acute Medicine (JAAM criteria) [4]; DIC was defined as a total score of ≥4.

### Treatment of *R. tigrinus* bites

The antivenom used against *R. tigrinus* bites was experimentally manufactured [1]. Severe adverse effects exclusively refer to anaphylactic shock in which the patient is at a risk of death because of antivenom administration.

### Data collection

The following parameters were recorded: age, gender, date of injury, clinical symptoms, laboratory data, and DIC score as well as treatment-related factors and the outcomes including hospital mortality and renal failure requiring hemodialysis.

### Outcome measures

The primary endpoint of the present study was to determine the association between antivenom administration and hospital mortality. The secondary outcome was to determine the association between antivenom administration and renal failure requiring hemodialysis after the acute phase of injury.

### Primary data analysis

Statistical analysis was performed using JMP version 11 (SAS, Cary, NC, USA). Patient characteristics, treatment-related factors, and outcomes were compared between the antivenom group and the without antivenom group using Mann–Whitney *U* test and *χ*^2^ test or, where appropriate, the Fisher exact test for categorical variables. *P* values of ≤0.05 alpha were considered statistically significant.

## Results

### Demographic data and clinical characteristics of all study patients

Over the 43-year study period, 34 patients were identified; the patient characteristics are summarized in Table 1. We further analyzed 25 cases from the previous study [3]. All patients, except for one, were male, with a median age of 37.5 years. On admission, the median levels of fibrinogen and fibrinogen degradation products (FDPs) were 35 mg/dL and 200 μg/mL, respectively, and platelet counts were 107,000/mm^3^. The mean DIC score was 5.

**Table 1:** **Population characteristics,** ***n*** **= 34**

**Population characteristics**	**Values**
Age (years)	37.5 (43.8)
Gender, male, *n* (%)	33 (97.1)
Date of getting injury (year)	
1973–1999	25 (73.5)
2000–2013	9 (26.5)
Clinical symptoms	
Nasal bleeding, *n* (%)	4 (11.8)
Gum bleeding, *n* (%)	15 (44.1)
Bleeding from the bite sites, *n* (%)	27 (79.4)
Headache, *n* (%)	6 (17.6)
Laboratory data	
Platelet counts (×10^4^/mm^3^)	10.7 (10.4)
Fibrinogen (mg/dL)	35 (30)
PT-INR	5 (4.38)
FDP (μg/mL)	200 (180)
DIC score	5(3)
Treatment	
Heparin, n (%)	14 (41.2)
FFP, n (%)	8 (25.0)
PE, n (%)	4 (11.8)
Antivenom, n (%)	19 (55.9)
Time interval between getting Yamakagashi bites and antivenom administration (h)	32 (31)
Severe adverse effects related to antivenom	0 (0)
Outcome	
Mortality, *n* (%)	4 (11.8)
Hospital stay	9.5 (9.5)
Renal failure requiring hemodialysis, *n* (%)	7 (20.6)

Data are presented as median (interquartile, IQR) for continuous variables and *n* (percentage) for categorical variables. *PT-INR* prothrombin time international ratio, *FDP* fibrinogen degradation products, *DIC* disseminated intravascular coagulation, *FFP* fresh frozen plasma, *PE* plasma exchange, *SD* standard deviation.

Antivenom was administered to 19 patients, and the median interval between bite and antivenom administration was 32 h. No apparent adverse effects were observed. DIC was treated with heparin in 14 patients. Seven patients developed renal failure requiring hemodialysis after the acute phase of the injury, and the in-hospital mortality rate for all the patients was 11.8%.

### Comparison of clinical characteristics between the antivenom and without antivenom groups

The comparison of clinical characteristics between the antivenom and without antivenom groups is summarized in Table 2. Baseline characteristics and laboratory data were not significantly different between the two groups.

**Table 2:** **Comparison between the antivenom and the without antivenom groups**

**Characteristics**	**Antivenom group (** ***n*** **= 19)**	**Without antivenom group (** ***n*** **= 15)**	***P*** **value**
Age (years)	37 (40)	43 (50)	0.93
Gender, male, *n* (%)	18 (94.7)	15 (100)	1.00
Date of injury, year (2000–2013), *n* (%)	7 (36.8)	2 (13.3)	0.24
Clinical symptoms			
Nasal bleeding, *n* (%)	1 (5.3)	3 (20.0)	0.07
Gum bleeding, *n* (%)	8 (42.1)	7 (46.7)	1.00
Bleeding from the bite sites, *n* (%)	16 (84.2)	11 (73.3)	0.67
Headache, *n* (%)	3 (15.8)	3 (20.0)	1.00
Laboratory data			
Platelet counts (×10^4^/mm^3^)	12.5 (10.1)	7.9 (11.6)	0.21
Fibrinogen (mg/dL)	42.5 (20)	31 (43)	0.34
PT-INR	5.84 (4.24)	2.81 (4.11)	0.1
FDP (μg/mL)	236 (185)	160 (214)	0.06
DIC score	5 (4)	4.5 (3)	0.6
Treatment			
Heparin, *n* (%)	4 (21.1)	10 (66.7)	0.01
FFP, *n* (%)	3 (15.8)	5 (38.5)	0.22
PE	1 (5.3)	3 (20.0)	0.30

Data are presented as median (interquartile, IQR) for continuous variables and *n* (percentage) for categorical variables. *PT-INR* prothrombin time international ratio, *FDP* fibrinogen degradation products, *DIC* disseminated intravascular coagulation, *FFP* fresh frozen plasma, *PE* plasma exchange, *SD* standard deviation.

Heparin use in the antivenom group was significantly lower than that in the without antivenom group (21.1% vs. 66.7%, *P* = 0.01).

### Correlations between antivenom administration and outcomes

**Figure 1:**
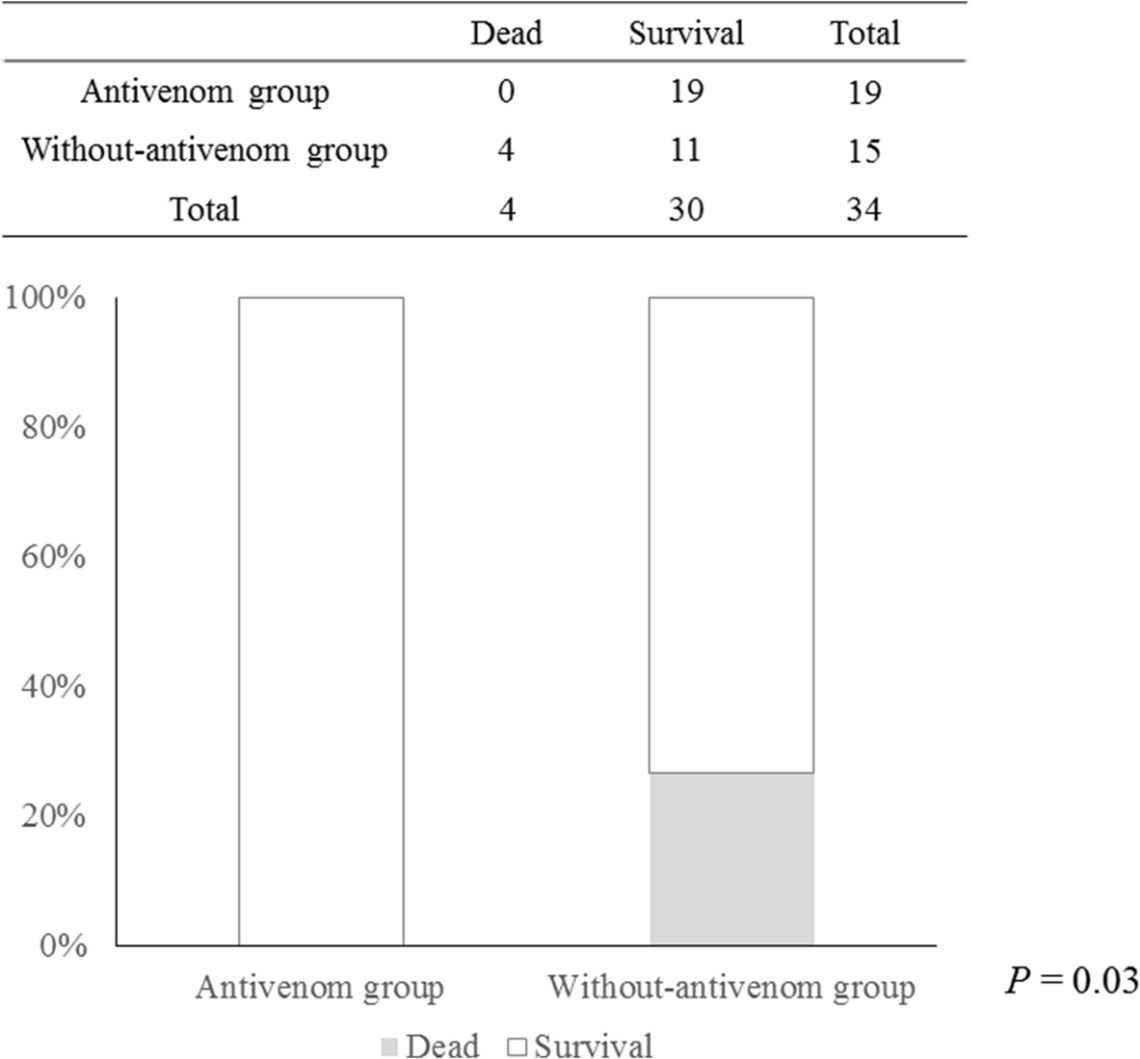
**Comparison of hospital mortality between the antivenom and the without-antivenom groups.** Hospital mortality in the antivenom group was significantly better than that in the without antivenom group (0% vs. 26.7%, *P* = 0.03).

**Figure 2:**
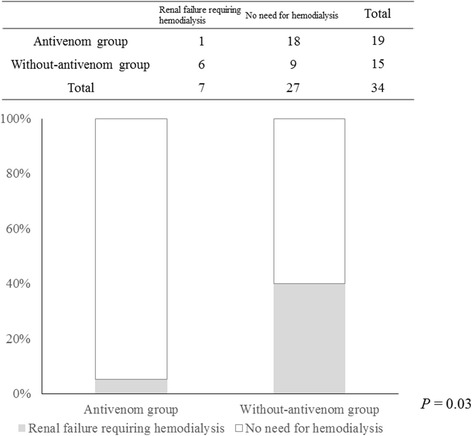
**Comparison of the number of patients developing renal failure requiring hemodialysis between the antivenom and the without antivenom groups.** The number of patients developing renal failure requiring hemodialysis was significantly lower in the antivenom group (5.3% vs. 40.0%, *P* = 0.03).

Hospital mortality in the antivenom group was significantly better than that in the without antivenom group (0% vs. 26.7%, *P* = 0.03) (Figure 1). Moreover, the number of patients developing renal failure requiring hemodialysis was significantly lower in the antivenom group (5.3% vs. 40.0%, *P* = 0.03) (Figure 2).

## Discussion

In the present study, we demonstrated that hospital mortality and the number of renal failure requiring hemodialysis following the acute phase of *R. tigrinus* bites were significantly better in patients receiving antivenom than in those not receiving antivenom. Previously, we demonstrated that the pathophysiology of *R. tigrinus* bites involves DIC with the fibrinolytic phenotype [3]. However, it seems that this DIC with fibrinolysis phenomenon does not persist throughout hospitalization and may be limited to the acute injury phase. The present survey revealed that in the acute phase, patients developed DIC with the fibrinolytic phenotype; however, 40% of patients without antivenom developed renal failure requiring hemodialysis in the later phase of the injury. Renal pathology has revealed that glomerular fibrin thrombi and tubular necrosis are responsible for renal failure associated with *R. tigrinus* bites [5]. Indeed, Gando et al. reported that 24 to 48 h after severe traumatic injury, DIC with the fibrinolytic phenotype changes to DIC with the thrombotic phenotype, which can result in the fatal multiple organ dysfunction syndromes (MODS) [6,7].

Gando et al. argued that the guiding principal in the treatment of DIC is the specific and vigorous treatment of the underlying disorder [6]. Considering our current understanding of the pathophysiology of *R. tigrinus* bites, it is obvious that managing DIC with heparin is contraindicated in the acute phase because patients develop bleeding manifestations [8]. On the other hand, antivenom represents a specific, definitive, and effective treatment in this phase. It appeared that administering *R. tigrinus* antivenom following bites can lead to complete clinical recovery without progression to MODS, even in the presence of severe DIC. Thus, antivenom effectively treats the acute symptoms and can prevent disease progression. If there is appropriate preparedness for anaphylaxis, antivenom should be used in patients with *R. tigrinus* bites.

A major adverse effect of antivenom is serum sickness disease, which usually occurred in 4–10 days after administration of antivenom [9]. Rashes, itching, joint pain, fever, lymphadenopathy, malaise, and renal failure are typical symptoms [9,10]. Because the number of patients developing renal failure requiring hemodialysis was significantly lower in the antivenom group, the close association between antivenom administration and renal failure was not considered. In the present study, although the numbers in the present survey are still too low to make any comprehensive assessment, the initial anaphylactic reaction rate was also lower than the 2.4%–9% rate observed with *G. blomhoffii* antivenom [11,12].

There are many limitations to the present study. Notably, the present study had a retrospective design and a relatively small sample size. Selection bias may also have been an issue because only cases reported to our center were used, and many cases may have remained undiagnosed or misdiagnosed because of the unfamiliar symptoms presented by this rare snakebite. Finally, because tissue plasminogen activator (t-PA) was not evaluated, the primary activation of fibrinogenolysis remains unclear. Furthermore, plasminogen activator inhibitor-1 (PAI-1), which induces the suppression of fibrinolysis, was not evaluated. Further study is required to clarify the pathophysiology of *R. tigrinus* bites.

## Conclusions

In our small retrospective study, antivenom administration was likely to be effective in the management of *R. tigrinus* bites. Apparently, further research is required to confirm its efficacy.
